# The impact of agricultural support and protection subsidy policy on grain production efficiency: A case study of China

**DOI:** 10.1371/journal.pone.0337407

**Published:** 2025-12-16

**Authors:** Yuan Tian, Yuxi Zhou

**Affiliations:** School of Economics and Management, Shandong Agricultural University, Taian, China; Policy Resaerch Institute, Government of Nepal, NEPAL

## Abstract

Based on panel data from 31 Chinese provinces from 2012 to 2022, this study employs a difference-in-difference method to assess the impact of the agricultural support and protection subsidy (ASPS) policy on grain production efficiency. The results indicate that the ASPS policy has a significant positive impact on grain production efficiency, and the results remain robust through multiple robustness tests. Furthermore, heterogeneity analysis reveals that the policy’s impact varies substantially across different regions and types of grain crops. Mechanism analysis further demonstrates that the ASPS policy enhance grain production efficiency by improving the grain cultivation areas and productive inputs. Consequently, we propose targeted policy recommendations, including enhancing the intensity and precision of subsidies, implementing differentiated subsidy approaches, and utilizing subsidies to stimulate dual efficiencies in both scale and investment.

## 1. Introduction

Food security constitutes a fundamental pillar of national security infrastructure. In order to promote grain output and ensure food security, the Chinese government launched a comprehensive agricultural subsidy system in 2004. This system included seed subsidies, direct grain cultivation subsidies, and comprehensive agricultural support funds. These initiatives have significantly contributed to increased grain production [1]. However, as the landscape of agricultural and rural development evolves, the limitations of these subsidies have become increasingly apparent: First, the subsidies lack precision, as they are distributed based on contracted land area rather than actual cultivated land area. Consequently, primary agricultural producers engaged in large-scale cultivation, including commercial grain growers and family farms frequently encounter barriers to accessing proportionate subsidy benefits. Second, the subsidy mechanism constrains the scale of grain farming operations. Subsidies are distributed based on contracted land holdings, and after land transfers, the actual cultivators can not receive the subsidies. Furthermore, agricultural subsidies have gradually evolved into universal income support for farmers rather than production incentives, resulting in a significant diminishing marginal effect of the subsidies [2]. Therefore, in order to adapt to the new situation of agricultural production and guarantee national food security, it is urgent to implement agricultural subsidy policies reforms.

In view of the above background, since 2015, the government of China has initiated a pilot reform of its agricultural subsidy policy. Then the reform began to be rolled out nationwide in 2016. The core initiative of this round of reform is to unify and integrate the original three agricultural subsidies into an agricultural support and protection subsidy (ASPS). The reformed ASPS policy directly supports grain producers and ensures the accuracy and effectiveness of the subsidies, enhancing the precision of subsidies. This enables the entities actually engaged in grain production to receive greater subsidies, establishing a virtuous mechanism where “those who grow more grain receive priority support”. Furthermore, unlike previous subsidy schemes, the reformed ASPS policy focuses on supporting the conservation of arable land fertility and promoting moderate-scale grain operations. This approach not only advances large-scale grain production but also fosters green agricultural development. Current literature primarily investigates the policy’s effectiveness in boosting grain output and raising agricultural household earnings [3]. In general, the source of agricultural economic growth can be summarized in two fundamental aspects: the augmentation of production factor inputs and the improvement of production efficiency. Within the context of continuous economic development, as the limitations of relying solely on factor accumulation to drive economic growth have become increasingly apparent, the enhancement of production efficiency has emerged as the crucial catalyst for promoting agricultural economic advancement [4,5]. Consequently, conducting in-depth research and scientific evaluation on the impact of ASPS on grain production efficiency holds significant importance for policy optimization and adjustment.

So, does the ASPS policy have any impact on the grain production efficiency? What is the impact? Based on these questions, this paper uses the panel data of 31 provinces (municipalities and autonomous regions) in China from 2012 to 2022 to assess the impact of ASPS policy on grain production efficiency with the help of difference-in-differences (DID) model. And then this paper carries out a mechanism analysis to explore the mechanism of ASPS policy affecting grain production efficiency, with a view to providing scientific basis for promoting grain production, guaranteeing food security and improving agricultural subsidy policies.

Compared with extant literature, the contributions and innovations of this paper can be summarized as follows. First, China’s agricultural subsidy reform provides a unique experimental setting, and the transition between old and new policies also represents a test of a theoretical hypothesis: Can more targeted subsidies enhance production efficiency? This study focuses on examining the policy effects during the period following agricultural subsidy reform. It not only verifies effectiveness but also reveals how the effects materialize through mechanism analysis, uncovering heterogeneity in outcomes. Second, while existing literature extensively discusses subsidies’ impact on output and income, research on their effects on production efficiency—a crucial indicator for measuring resource allocation—remains relatively scarce. This paper enriches and complements research in this field. Finally, this study provides empirical evidence and practical insights from China for refining agricultural subsidy policies globally.

The paper proceeds as follows: Section 2 surveys extant literature on agricultural subsidy policy and ASPS policy. Section 3 presents the research methodology. Then the findings and their interpretation are detailed in Section 4. Section 5 demonstrates further discussion including heterogeneity analysis and mechanism analysis. Concluding remarks with policy implications are presented in Section 6.

## 2. Literature review

Agricultural subsidy policy is an important measure taken by countries around the world to protect and promote agricultural production, and the policy has profoundly affected agricultural production and farmers’ lives. Based on extensive existing literature, this study summarizes the following perspectives.

### 2.1. Research progress on the impact of agricultural subsidy policies on agricultural production

On the one hand, several scholars think that agricultural subsidy policy has a positive influence on agricultural production. Mamun proves that agricultural subsidies contribute to the growth of agricultural productivity in 42 countries around the world [6]. Garrone et al. show that agricultural subsidies in the European Union increase agricultural labor productivity, with differential impacts observed across subsidy instruments [7]. Kirwan finds that agricultural subsidies have a positive impact on slowing down the contraction of farm size through examining U.S. farms [8]. Yi et al. argue that agricultural subsidy policy play a positive role in farmers’ production behavior, particularly enhancing low-income farmers’ willingness to expand grain cultivation through income improvement mechanisms [9]. The study of Guo et al. shows that agricultural subsidies lead to a reduction in the use of fertilizers by farmers growing rice [10]. Qian et al. provide evidence of subsidy policies’ beneficial impacts on grain market prices in China [11].

On the other hand, some scholars believe that agricultural subsidy policies have a negative or insignificant impact on agricultural production. Gebeyehu and Bedemo examine the impact of agricultural credit and subsidies on agricultural productivity in Ethiopia, and the results show that in the long term, the impact of credit is positive but subsidies negatively impact productivity [12]. Du et al. find that subsidies increase inputs of agricultural factors of production, leading to an increase in carbon emissions [13]. Kang and Suh argue that agricultural subsidies lead to increased grain losses in both the short and long term [14]. In addition, agricultural subsidy levels are decoupled from actual cultivated land area. And low subsidy amounts fail to promote farmers’ incentives to grow grain [15]. From the perspective of differences in farmers’ incomes, subsidies do not significantly promote total factor productivity in wheat, and this effect does not differ between high or low income farmers [16]. Huang et al. conclude that although agricultural subsidies promote farmers’ income growth, they have no significant impact on grain production [17].

### 2.2. Research progress on the evaluation of ASPS in China

Since the initiation of pilot reforms to China’s ASPS policy in 2015, scholars have commenced comprehensive investigations into its impacts. The research centers on the influence of ASPS on grain production, farmers’ behavior and ecological environment. Firstly, the influence of ASPS on grain production is mainly reflected in the area of grain cultivation and grain yield, etc. Fan et al. demonstrate that these subsidies expand grain crop cultivation by incentivizing land rental and increasing grain planting allocation [18]. Complementarily, Zhu et al. establish ASPS’s positive role in elevating grain yields and enhancing crop quality [19]. Secondly, when scholars explore the impact of ASPS on farmers’ behavior, they mainly focus on how it affects farmers’ planting choices and input behaviors, and deeply analyze the internal mechanisms. For example, Xu et al. use data from rural fixed observation sites in China to explore the specific effects of ASPS on scale farmers. Specifically, ASPS is conductive to accelerating land transfers and expanding the scale of grain production [20]. Long and Li explore the positive effects of ASPS on grain farmers’ increased production and increased incomes, using data from 512 large-scale farmers in Hunan Province, China. And the results demonstrate that the influence of the policy is more significant for large-scale farmers with less than 350 acres of planting area [21]. Finally, the influence of ASPS on the ecological environment mainly unfolds from the protection of arable land resources and the control of surface source pollution. ASPS has a facilitating effect on the transfer of arable land, which effectively inhibits the idleness of arable land and strengthens the protection of arable land resource [22]. However, ASPS have no positive influence on the prevention and control of agricultural surface source pollution, but instead stimulates farmers to increase the amount of pesticide application [23]. In addition, some scholars have measured and evaluated the efficiency of ASPS. For example, Wang measured the efficiency of the ASPS policy in five cities in northern Jiangsu Province, China. And the results demonstrate that although the technical efficiency of the policy basically reaches the optimal state, its promotion of large-scale production of grain is still insufficient at the level of scale efficiency and comprehensive efficiency [24].

Based on the relevant literature, it can be found that the current results on the impact of subsidy policies on agricultural production are rich, which lays the foundation for this study. However, scholars have not yet reached a consistent conclusion due to the different research perspectives and contents. In the literature on the assessment of ASPS policy, investigations predominantly examine its effect on farmer income and output increases, with limited attention paid to ASPS implications for grain production efficiency. Furthermore, most of the literature explores the influence of policies on grain production before the agricultural subsidy reform, and there is not enough research on the ASPS policy after the reform. Therefore, this paper focuses on the effectiveness of the implementation of the reformed ASPS, using the DID model to assess the influence on grain production efficiency.

## 3. Research design

### 3.1. Theoretical analysis

#### 3.1.1. The direct impact of ASPS on grain production efficiency.

According to the principle of utility maximization, farmers will adjust their production behaviors after receiving ASPS, thereby influencing grain production efficiency. First of all, ASPS will increase investment in agricultural production. In a resource-constrained agricultural production environment, ASPS will alleviate farmers’ financial constraints, enabling them to increase production inputs, such as expanding fertilizer use or adjusting input structures (e.g., adopting organic fertilizers), which can effectively improve production efficiency. Secondly, ASPS will also prompt farmers to make investment decision adjustments. For example, subsidy recipients demonstrate higher probabilities of expanding grain cultivation scale with the incentive of subsidies. And large-scale operation can be more effective to improve the grain production efficiency. Finally, ASPS help promote agricultural mechanization. Grain farmers frequently allocate subsidy funds to buy agricultural machinery services or advanced agricultural equipment, thereby upgrading production technologies, increasing yields, and ultimately enhancing production efficiency [25]. Furthermore, ASPS can boost farmers’ grain cultivation incentives. By increasing farmers’ income, the subsidies facilitate participation in technical training and continuing education programs, improving cultivation skills and reinforcing production motivation [26]. The subsidies also provide financial support for operational expansion, encouraging farmers to acquire additional land and achieve scale economies, which collectively strengthen farmers’ commitment to grain production [27]. In summary, both the improvement can effectively promote grain production efficiency. Consequently, this paper proposes Hypothesis 1.

H1: ASPS have significant positive impacts on enhancing grain production efficiency.

#### 3.1.2. Mechanism analysis of ASPS affecting grain production efficiency.

Low grain production efficiency is often caused by many reasons, and some scholars have pointed out that it is mainly to the small-scale agricultural cultivation areas and low productive inputs in developing countries [28]. Therefore, this paper mainly analyzes the intrinsic mechanism of ASPS on grain production efficiency from the two aspects of grain cultivation scale and productive inputs. Specifically, first, the subsidy policy enhances grain production efficiency by promoting the expansion of grain planting areas. After the reform of ASPS, the subsidies are allocated based on planted acreage, adhering to the principle of “priority support for larger producers.” Recipients can utilize this financial assistance to expand grain planting area, fostering economies of scale that enhance efficiency. In addition, this subsidy standard may also incentivize grain farmers to carry out land transfer or change the planting structure to expand the scale of grain cultivation area [4], thus forming scale effects that improve output per unit. Second, ASPS directly stimulates grain farmers to increase productive inputs, such as improving production technology and introducing advanced equipment. First of all, ASPS alleviate farmers’ financial constraints, enabling increased investment in production factors and technological upgrades. Secondly, subsidies increase the probability of farmers to use agricultural machinery and equipment. In the short term, farmers can use the subsidy funds to pay for the operating costs of machinery, and then use mechanization to promote the significant increase in grain production efficiency. In the long term, the subsidy policy will enhance the willingness and possibility of farmers to purchase large agricultural machinery, which will also bring significant positive effects on grain production efficiency. Overall, this paper proposes Hypothesis 2.

H2: ASPS will improve grain production efficiency by expanding the scale of grain cultivation area and increasing productive inputs.

### 3.2. Model construction

#### 3.2.1. Data envelopment analysis.

Data Envelopment Analysis (DEA), as a quantitative tool for assessing the relative efficacy of Decision Making Units (DMUs), was initially proposed by scholars such as Charnes et al in 1978 [29]. And since then, the methodology has been widely adopted and utilized in the assessment of efficiency in several fields. In this paper, we use an output-oriented DEA model to accurately determine the grain production efficiency under the assumption of variable returns to scale. And grain production efficiency is categorized into three dimensions: technical efficiency (TE), pure technical efficiency (PTE) and scale efficiency (SE). Specifically, TE evaluates the integrated capacity of DMUs in terms of resource allocation, PTE focuses on the direct contribution of management and technological innovation to production efficiency, and SE specifically measures the impact of production scale on the level of efficiency. In this study, TE provides a comprehensive measure of overall resource allocation performance and serves as a direct objective for evaluating the overall effectiveness of policies. Therefore, TE is selected as the dependent variable. PTE reflects the level of management and technology in the grain production process, while SE reflects the rationality of the scale of grain production and operation. Assuming that there are i(i=1,2,...,k) DMUs, each with $r(x_{i1},x_{i2},...,x_{ir})$ input factors and $s(y_{i1},y_{i2},...,y_{is})$ output factors, the specific formula is as follows [30].


$$\begin{array}{c} Min[\theta -\varepsilon (e_{1}^{T}s^{+}+e_{2}^{T}s^{-})nonumber\\ s.t.\left\{ \begin{array}{c} \sum_{i=1}^{k}\lambda_{i}x_{i}+s^{-}=\theta X_{0}\\ \sum_{i=1}^{k}\lambda_{i}y_{i}-s^{+}=Y_{0}\\ \sum_{i=1}^{k}\lambda_{i}=1\\ \lambda_{i}\geq 0,i=1,2,...,k\\ s^{-}\geq 0,s^{+}\geq 0 \end{array}\right. \end{array}$$ (1)

Where θ is the comprehensive efficiency of DMUs, and θ=1 indicates that the combined efficiency is optimal. $s^{+},\;s^{-}$ are the relaxation variable. ε represents the value of the non-Archimedean infinitesimal quantity parameter. $e_{\text{1}}^{T},\;e_{\text{2}}^{T}$ are the summation vector. $x_{i},\;y_{i}$ denote the inputs and outputs of i th DMU. $\lambda_{i}$ denotes the weights of DMUs.

#### 3.2.2. Basic regression model.

In order to investigate the impact of ASPS on grain production efficiency, this study regards the reform of ASPS policy as a quasi-natural experiment, and constructs a multi-period DID model. Since the ASPS reform was piloted in five provinces in 2015 and implemented nationwide in 2016, we select a multi-period DID model with all individuals entering the treatment group to evaluate the policy’s effects [23]. Referring to the study of Beck et al. [31], the following model is constructed:


$$TE_{it}=\alpha +\beta Policy_{it}+\delta X_{it}+\mu_{i}+\gamma_{t}+\varepsilon_{it}$$ (2)

Where $TE_{it}$ denotes the grain production technical efficiency of the i th province in t period. $Policy_{it}$ is a dummy variable for the implementation of ASPS policy, which is assigned the value of 1 if province i has carried out the reform of ASPS during the t period, otherwise it is 0. $X_{it}$ represents the control variables. α is a constant term. β and δ are the parameters to be estimated, and β is the coefficient of the core explanatory variable, which represents the net effect of ASPS on grain production efficiency. $\mu_{i}$ denotes individual fixed effects, and $\gamma_{t}$ denotes time fixed effects. $\varepsilon_{it}$ is a random error term.

### 3.3. Variable selection

#### 3.3.1. Explanatory variables.

In this study, the TE of grain production of each province is selected as the explanatory variable. Firstly, establishing an indicator system. Referring to existing studies on grain production efficiency [32–34], and based on the principles of data availability and scientificity of indicators, an indicator system is constructed in Table 1. In view of the fact that some input resources are not clearly distinguished between grain crops and other crops in the statistics, for ensuring the accuracy of grain production input estimation, this study adopts the weight coefficients method to separate the inputs related to grain production [35,36]. The specific operation is as follows: A = grain sown area/total crops sown area, B = agricultural output value/total output value of agriculture, forestry, animal husbandry and fishery. Secondly, using MaxDEA software to calculate grain production efficiency, given the constraints on China’s agricultural land and water resources, farmers’ objectives are more likely to be minimizing costs by reducing inputs at a given output level. Therefore, this study adopts an input-oriented model that better aligns with the decision-making conditions of Chinese grain producers. Finally, efficiency is broken down into three dimensions: total efficiency (TE), pure technical efficiency (PTE), and scale efficiency (SE), with TE selected as the dependent variable.

**Table 1:** Indicator system for evaluating grain production efficiency.

Variable Category	Variable Name	Variable Definition	Variable Unit	Data Sources
Input variables	Land input	Grain sown area	10^3^ hm²	China Rural Statistical Yearbook
	Mechanical input	Gross power of agricultural machinery*A	10^4^ kW	China Rural Statistical Yearbook
	Irrigation input	Effective irrigated area*A	10^3^ hm²	China Rural Statistical Yearbook
	Fertilizer input	Fertilizer application*A	10^4^ t	China Rural Statistical Yearbook
	Labor input	Number of employees in the primary sector*B*A	Ten thousand people	China Statistical Yearbook
Output variables	Grain production	Total grain production	10^4^ t	China Rural Statistical Yearbook

#### 3.3.2. Core explanatory variables.

The core explanatory variable is ASPS policy dummy variable (Policy), which is assigned the value of 0 before the implementation and the value of 1 after the implementation. The ASPS policy began to be reformed on a pilot basis in 2015 in five provinces: Shandong, Zhejiang, Anhui, Hunan, and Sichuan, and it has been implemented nationwide since 2016. Therefore, from 2012 to 2014, the Policy is set to 0 for all provinces. Starting in 2015, the Policy is set to 1 for five provinces: Shandong, Zhejiang, Anhui, Hunan, and Sichuan. Beginning in 2016, the Policy is set to 1 for all other provinces.

#### 3.3.3. Control variables.

Referring to the related research [25,37,38], we select some control variables to ensure the accuracy and validity of the regression as far as possible. (1) Industrial structure (Is), reflecting the contribution degree of the primary industry output value to the total output value of the region. (2) Agricultural machinery density (Amd), reflecting the agricultural machinery level and the agricultural modernization level. (3) Percentage of financial expenditure on agriculture (Fea), reflecting the degree of governmental support for agricultural production and life, etc. (4) Agricultural planting structure (Aps), reflecting the proportion of planting of grain crops. (5) Grain crops damaged area (Fda), reflecting the extent of grain crop damage. (6) Effective irrigation rate (Eir), reflecting the irrigation conditions in a particular region.

#### 3.3.4. Mechanism variables.

According to the previous analysis, the ASPS may affect the grain production efficiency through the grain cultivation scale and productive inputs. In this paper, we select two mechanism variables, the grain cultivation scale, denoted by the per agricultural household grain cultivation area (Pah), and productive inputs, denoted by the total power of agricultural machinery (Pam).

The meaning and descriptive statistics of the variables are shown in Table 2.

**Table 2:** Descriptive statistics of variables.

Variable name	Variable Code	Variable Definition	Variable unit	Data Sources	Observed value	Average value	Standard deviation
Grain production technical efficiency	TE	Measured using DEA	%	\	341	0.851	0.122
Policy dummy variables	Policy	0 before policy implementation, 1 after policy implementation	\	\	341	0.651	0.477
Industrial structure	Is	Primary sector output/Gross regional output	%	China Statistical Yearbook	341	0.098	0.053
Agricultural machinery density	Amd	Total power of agricultural machinery/Total sown area of crops	10kW-hm^-2^	China Rural Statistical Yearbook	341	0.689	0.346
Percentage of financial expenditure on agriculture	Fea	Expenditure on agriculture, forestry and water affairs/Total fiscal expenditure	%	Statistical Yearbook of Each Province	341	0.116	0.035
Agricultural planting structure	Aps	Area sown in grain crops/Total area sown in crops	%	China Rural Statistical Yearbook	341	0.650	0.144
Grain crops damaged Area	Fda	Damaged area* (Area sown in grain crops/Total area sown in crops)	10^3^ hm^2^	China Rural Statistical Yearbook	341	480.233	589.626
Effective irrigation rate	Eir	Effective irrigated area/ Total area sown in crops	%	China Rural Statistical Yearbook	341	0.452	0.189
Per agricultural household grain cultivation area	Pah	Area sown in grain crops/ Number of rural households	10^3^hm^2^/10^4^ households	China Statistical Yearbook	341	6.404	5.140
Total power of agricultural machinery	Pam	Total power of agricultural machinery	10^4^ kW	China Rural Statistical Yearbook	341	3342.996	2903.767
Rural non-labor force population	IV	Rural non-labor force population	10^4^ people	Statistical Yearbook of Each Province	341	6.019	1.023

### 3.4. Data sources

This study utilizes data spanning 2012–2022, covering mainland China’s 31 provinces (autonomous regions and municipalities). Primary sources include the China Statistical Yearbook and the China Rural Statistical Yearbook, supplemented by regional statistical records. Hong Kong, Macao, and Taiwan are excluded from the analysis due to extensive data gaps.

## 4. Results and analysis

### 4.1. Results of the basic regression model

Model (2) is applied to estimate the impact of ASPS on grain production efficiency, and Table 3 demonstrates the regression results. Column (1) represents the regression results without adding other variables and only adding policy dummy variables. Columns (2) to (7) represent the effects of gradually adding Is, Amd, lnFea, Aps, lnFda, Eir, respectively. Table 3 demonstrates that the impact of ASPS on grain production efficiency passes the significance test at the 1% level under either model, reflecting that the model estimation results are well robust. At the same time, the coefficients of policy variables are positive, indicating that ASPS policy can significantly improve grain production efficiency. On average, the ASPS policy can improve grain production efficiency by 2.74% without considering other factors. As for the control variables, Amd and Fda exhibit statistically adverse effects, while Aps shows beneficial impacts, and remaining variables lack statistical significance. Given the significant differences in grain production conditions across provinces, the lack of agricultural mechanization and the limitations of input scale often limit the comprehensive and effective use of grain production materials, which in turn constitutes a major obstacle to improve grain production efficiency. In addition, Fda’s negative influence stems primarily from flood and drought disasters that cause substantial output reductions. The positive association with Aps likely reflects economic incentives—expanded cultivation areas motivate greater farmer investments to obtain more economic benefits, thereby boosting productivity.

**Table 3:** Impact of ASPS policy on grain production efficiency.

Variable	(1)	(2)	(3)	(4)	(5)	(6)	(7)
Policy	0.0285 ^***^ (3.8350)	0.0284 ^***^ (3.8227)	0.0279 ^***^ (4.021)	0.0255 ^***^ (4.0173)	0.0266 ^***^ (3.3833)	0.0270 ^***^ (3.3123)	0.0278 ^***^ (3.3238)
Is		0.3644 (0.7549)	0.2553 (0.5541)	0.2532 (0.5260)	0.2686 (0.5099)	0.2685 (0.5297)	0.3098 (0.5847)
Amd			−0.0645^**^ (−2.1029)	−0.0657^**^ (−2.1423)	−0.0573^**^ (−2.3200)	−0.0622^**^ (−2.5274)	−0.0542^**^ (−2.0678)
Fea				0.3295 (1.1225)	0.4098 (1.388)	0.4550 (1.5062)	0.4053 (1.425)
Aps					0.1961 (1.5857)	0.2153^*^ (1.7351)	0.2237^*^ (1.7929)
lnFda						−0.0059^*^ (−1.7940)	−0.0065^*^ (−1.8912)
Eir							−0.0659 (−1.1299)
Individual fixed	Yes	Yes	Yes	Yes	Yes	Yes	Yes
Time fixed	Yes	Yes	Yes	Yes	Yes	Yes	Yes
Constant	0.8327 ^***^ (172.3405)	0.7971 ^***^ (16.4169)	0.8525 ^***^ (16.9509)	0.8169 ^***^ (12.7714)	0.6722 ^***^ (5.5615)	0.6882 ^***^ (5.9811)	0.7114 ^***^ (6.5983)
Observation	341	341	341	341	341	341	341
R^2^	0.9098	0.9100	0.9126	0.9133	0.9154	0.9161	0.9162

Note: Values in parentheses are standard errors, ***, **, and * indicate significance at the 1%, 5%, and 10%

significance levels, respectively. The same applies below.

### 4.2. Parallel trend test

The parallel trend assumption is fundamental to DID estimation, requiring comparable pre-treatment outcome trajectories across regions. Drawing upon Beck et al.‘s methodology, we implement the following verification model. [31].


$$TE_{it}=\alpha +\sum_{t=-3}^{4}\beta_{t}Policy_{it}+\delta X_{it}+\mu_{i}+\gamma_{t}+\varepsilon_{it}$$ (3)

The variables and coefficients in the formula are consistent with those in Model(2). $\beta_{t}$ reflects the difference in grain production efficiency between provinces with and without the implementation of ASPS policies by the t th year of policy enactment.

**Fig 1:**
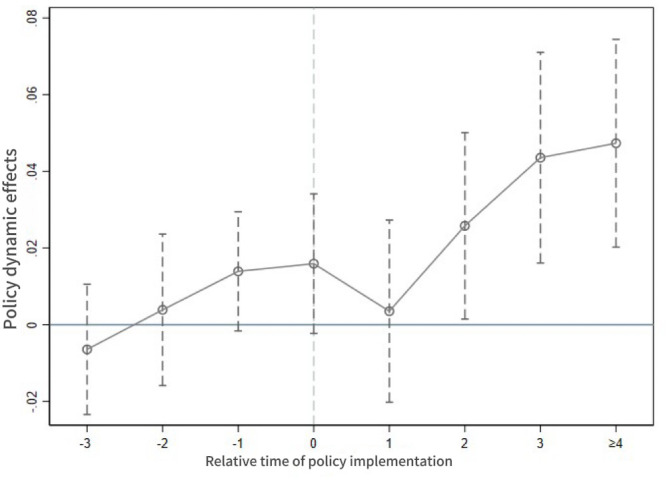
Parallel trend test results.

This study employs four periods before and after the implementation of ASPS policies to fit Model [3], with the fourth period preceding policy enactment designated as the baseline. Post-implementation data spanning four years are aggregated into the fourth period. Fig 1 illustrates the dynamic impact of the ASPS on the grain production efficiency, revealing statistically insignificant coefficients for all pre-intervention periods. So it satisfies the assumption of a parallel trend, and it is gradually significant in the 2nd period after the policy began to be implemented, which further indicates that the implementation of the policy continues to be effective while revealing a time-lagged impact pattern.

### 4.3. Placebo test

In order to preclude temporal effects from confounding provincial efficiency differences, the implementation time of ASPS policy is advanced by 2 years and 3 years respectively, which are denoted by $Policy^{-2}$ and $Policy^{-3}$. And then using Model (2) to test and outcomes are detailed in Table 4. Columns (1)-(2) present findings that the policy is advanced by 2 years, and columns (3)-(4) present findings that the policy is advanced by 3 years. Control variables are excluded in columns (1) and (3), but included in (2) and (4). The results demonstrate that the coefficient estimates of $Policy^{-2}$ and $Policy^{-3}$ both fail the test at the 10% significance level, confirming that there is no systematic difference in the time trend of grain production efficiency across provinces. Therefore, the estimation results and conclusions of Model (2) are robust, indicating the ASPS policy’s significant positive influence on grain production efficiency.

**Table 4:** Placebo test results.

Variable	2 years in advance		3 years in advance	
	(1)	(2)	(3)	(4)
Policy^-2^	0.0085 (0.8372)	0.0134 (1.4750)		
Policy^-3^			0.0251 (1.5659)	0.0256 (1.6020)
Other controls	Yes	Yes	Yes	Yes
Individual fixed	Yes	Yes	Yes	Yes
Time fixed	Yes	Yes	Yes	Yes
Constant	0.8442 ^***^ (100.4056)	0.7187 ^***^ (6.5839)	0.8281 ^***^ (55.9733)	0.6999 ^***^ (6.0992)
Observation	341	341	341	341
R^2^	0.9092	0.9157	0.9096	0.9161

### 4.4. Robustness tests

#### 4.4.1. Sample data screening.

In order to avoid the influence of individual extreme values on the results of the benchmark regression, the sample data are winsorized at 1% and 5% levels and re-estimated. Columns (1)-(2) of Table 5 confirm that ASPS maintains a statistically significant influence on grain production efficiency at the 1% level even after excluding extreme values, further reinforcing result stability.

**Table 5:** Robustness test results.

Variable	(1)	(2)	(3)	(4)	(5)	(6)	(7)	(8)
	winsorize at 1% level	winsorize at 5% level	Is	Amd	Fea	Aps	lnFda	Eir
Policy	0.0283 ^***^ (3.3852)	0.0264 ^***^ (3.1123)	−0.0002 (−0.0745)	−0.0177 (−0.4044)	0.0070 (1.5178)	−0.0068 (−0.3956)	0.1010 (0.4151)	0.0119 (1.4007)
Other controls	Yes	Yes	Yes	Yes	Yes	Yes	Yes	Yes
Individual fixed	Yes	Yes	Yes	Yes	Yes	Yes	Yes	Yes
Time fixed	Yes	Yes	Yes	Yes	Yes	Yes	Yes	Yes
Constant	0.7109 ^***^ (6.6218)	0.7074 ^***^ (6.8155)	0.0975 ^***^ (3.5791)	0.8265^*^ (1.7957)	0.1531 ^***^ (4.9557)	0.6662 ^***^ (6.4535)	3.5264 ^***^ (3.0712)	0.3518^**^ (2.0443)
Observation	341	341	341	341	341	341	341	341
R^2^	0.9164	0.9112	0.9792	0.9227	0.8981	0.9560	0.8700	0.9582

#### 4.4.2. Replacement of explanatory variables with control variables.

Robustness is further verified by sequentially replacing the explanatory variables with control variables. If the parameter estimates of the core explanatory variables are significant after replacement, the model fails the robustness test. Columns (3) to (8) of Table 5 present regression results using Is, Amd, Fea, Aps, lnFda, Eir as explained variables. None of the results pass the significance test, proving the robustness of the model.

#### 4.4.3 Instrumental Variables Test.

To avoid endogeneity issues arising from selection bias and reverse causality, this study employs instrumental variables and conducts a 2SLS estimation. Following the research by Gao and Yuan [38,39], the number of non-labor force members in rural households is selected as the instrumental variable for the ASPS reform. The primary reasons are as follows: First, the number of non-labor force members influences the scale of land contracting, while the subsidy amount after the ASPS reform is linked to this scale, indicating a close relationship between the two. Second, rural non-laborers do not directly participate in agricultural production and thus do not affect grain production efficiency.

The results of the instrumental variable test are shown in Table 6. The F-value exceeds 10, indicating that the model does not suffer from weak instrument issues. Furthermore, the regression coefficients are significantly larger than those estimated in Table 3, further confirming the robustness of the results.

**Table 6:** Instrument Variable Test.

Variable	First stage	Second stage
**Policy**	**TE**
Policy		0.1851^**^
		(2.44)
IV	0.1184^***^	
	(4.10)	
Other controls	Yes	
Individual fixed	Yes	
Time fixed	Yes	
Observation	341	
R^2^	0.1363	
F	16.845	

## 5. Further discussion

### 5.1. Heterogeneity analysis

#### 5.1.1. Heterogeneity analysis of different regions.

In order to examine potential regional variations in the implementation effect of ASPS policy, based on the national strategic planning for “grain production zones,” the sample is divided into major grain production regions, major grain consumption regions, and balance of production and consumption regions for heterogeneity analysis. Table 7 shows the estimation results. In major grain production regions, the coefficient estimate of Policy is 0.0202, which passes the test at the 5% significance level. In the major grain consumption regions, the coefficient estimate of Policy is 0.014, but fails the test at the 10% significance level. And in the balance of production and consumption regions, the coefficient estimate of Policy is 0.0305, which passes the test at the 10% significance level. These results indicate that ASPS effectiveness varies substantially across regional types. And the policy significantly promotes the grain production efficiency in the major grain production regions and the balance of production and consumption regions, but does not have a significant effect on major grain consumption regions. The possible reason for this is that the economies of the main grain-consumption regions, including Beijing, Shanghai and Guangdong, are relatively developed, and the low amount of ASPS cannot effectively improve the conditions of grain production, so the impact on grain production efficiency is not significant. The grain production efficiency in major grain consumption regions has not significantly improved, which also suggests that its agricultural function may differ from the goal of ensuring “production capacity.” This indirectly indicates that the policy orientation of ASPS subsidies in major grain production regions to “stabilize production capacity” is effective.

**Table 7:** Impact of ASPS policy on grain production efficiency in different regions.

Variable	Major grain production regions	Major grain consumption regions	Balance of production and consumption regions
Policy	0.0202^**^ (2.6435)	0.0140 (1.2849)	0.0305^*^ (2.1876)
Other controls	Yes	Yes	Yes
Individual fixed	Yes	Yes	Yes
Time fixed	Yes	Yes	Yes
Constant	0.9557 ^***^ (12.7232)	0.6100 ^***^ (4.5273)	0.8452 ^***^ (6.0737)
Observation	142	78	121
R^2^	0.1830	0.4524	0.1451

#### 5.1.2. Heterogeneity analysis of different types.

In order to assess differential effects of the ASPS policy across different grain types, this paper selects three grain crops: rice, wheat and corn, and Table 8 presents the results. The impact coefficient of ASPS policy on the production efficiency of rice is 0.0255, which passes the test at the 5% significance level. The impact coefficient on the production efficiency of wheat is −0.0161, which does not pass the test of significance. And the impact coefficient on the production efficiency of corn is 0.0482, which passes the test at the 10% significance level. Therefore, these findings confirm the differential effects of the ASPS policy across different grain types, as evidenced by the significant positive impact on the production efficiency of rice and corn, while there is no significant impact on the production efficiency of wheat. Potential explanations may involve the misalignment between subsidy disbursement timelines and critical wheat production phases. Specifically, subsidy funds might not have been allocated to wheat cultivation inputs due to delayed distribution that miss either the planting season or key growth stages. Furthermore, the stagnant technological progression in wheat production systems likely limits the subsidies’ effectiveness. Current agricultural subsidies appear insufficient to drive technological upgrading within the wheat industry, failing to stimulate either advancements in cultivation techniques or the supply chain development of innovative agricultural technologies. This aligns with Gao’s research findings [32], which revealed that income subsidies significantly boost total factor productivity for rice and corn. However, even when accounting for lag effects, income subsidies still have no significant impact on wheat’s total factor productivity.

**Table 8:** Impact of ASPS policy on grain production efficiency of different types.

Variable	Rice	Wheat	Corn
Policy	0.0255^**^ (2.2371)	−0.0161 (−1.0838)	0.0482^*^ (1.8445)
Other controls	Yes	Yes	Yes
Individual fixed	Yes	Yes	Yes
Time fixed	Yes	Yes	Yes
Constant	0.7992 ^***^ (6.4426)	1.0138 ^***^ (5.5688)	0.9546 ^***^ (7.0138)
Observation	341	341	341
R^2^	0.0154	0.1348	0.1561

### 5.2. Mechanism analysis

Based on the previous theoretical and empirical analysis, and in order to investigate the mechanism of ASPS policy on grain production efficiency, we use the Pah and lnPam variables to indicate the grain cultivation scale and productive inputs respectively. Firstly, the policy variables are regressed with the Pah and lnPam variables respectively, and Table 9 demonstrates the results of the mechanism analysis. Columns (1)-(2) indicate that the coefficient estimates of Policyare positive and pass the test at 1% and 10% significance levels, respectively, which indicates that the ASPS policy has increased the grain cultivation scale and productive inputs. And the columns (3)-(4) show that the effect of grain cultivation scale and productive inputs on grain production efficiency, which demonstrates the coefficient estimate of grain cultivation scale is 0.0057 and passes the test at 1% level of significance, and the coefficient estimate of productive inputs is 0.0432 and passes the test at 10% level of significance. These results confirm that ASPS policy contributes to grain production efficiency by increasing grain cultivation scale and productive inputs.

**Table 9:** Mechanism analysis of ASPS policy affecting grain production efficiency.

Variable	(1)	(2)	(3)	(4)
	Pah	lnPam	TE	TE
Policy	1.1468 ^***^ (7.4262)	0.0260 ^*^ (1.7992)		
Pah			0.0057 ^***^ (2.5868)	
lnPam				0.0432 ^*^ (1.6968)
Other controls	Yes	Yes	Yes	Yes
Individual fixed	Yes	Yes	Yes	Yes
Time fixed	Yes	Yes	Yes	Yes
Constant	−0.5014 ^*^ (−0.2992)	7.0837 ^***^ (45.1976)	0.8339 ^***^ (12.2551)	0.5370 ^***^ (2.7642)
Observation	341	341	341	341
R^2^	0.1956	0.6087	0.0602	0.0485

Results from the regional heterogeneity analysis indicate that ASPS has the strongest impact on boosting grain production efficiency in major grain production regions with a certain scale foundation. This suggests that while the ASPS reform has clarified subsidy targets, within China’s current agricultural context, subsidy policy design may need to focus more on incentivizing the expansion of grain production scale rather than merely subsidizing farmers’ incomes to significantly enhance grain production efficiency. Therefore, the precision of subsidies requires further improvement. Regarding the mechanism analysis, this study reveals at the macro level how ASPS achieves efficiency gains by influencing farmers’ micro-level decisions. Expanding grain cultivation area serves as a key mechanism, reflecting the micro-level transmission mechanism of ASPS. Combined with the heterogeneity analysis results, this provides strong evidence for the precise design of the policy.

## 6. Conclusions and policy recommendations

### 6.1. Conclusions

In this study, we verify the impact effect of ASPS policy on grain production efficiency using the panel data from 31 provinces (autonomous regions and municipalities) across China spanning 2012–2022. Specifically speaking, we first measure the grain production efficiency of each province (autonomous region and municipality) by using DEA model. And then based on the idea of quasi-natural experiments, DID model is used to assess the impact effect of ASPS policy on grain production efficiency. Furthermore, we conduct further discussions including heterogeneity analysis and mechanism analysis. There are three principal research findings of this study.

Firstly, the primary empirical analysis confirms that ASPS policy substantially enhances grain production efficiency, demonstrating an average 2.74% efficiency gain without considering the influence of other factors. This outcome persists through comprehensive robustness verification. Secondly, the heterogeneity analysis results reveal differential policy effectiveness across regions and grain crop types. Specifically, significant productivity gains from ASPS policy implementation emerge specifically in the main grain production regions and the balance of production and consumption regions, while the impact on the main grain consumption regions is not significant. And the heterogeneity analysis of crop types reveals that ASPS policy significantly improves the production efficiency of rice and corn, but has no effect on wheat. Thirdly, mechanistic examination identifies that the ASPS policy can significantly promote the improvement of grain production efficiency by expanding the grain cultivation scale and increasing the productive resource inputs, primarily manifested through expanding grain planting areas and enhancing agricultural machinery investment. It demonstrates the intrinsic mechanism by which ASPS policy affects grain production efficiency.

### 6.2. Policy recommendations

These findings demonstrate ASPS’s efficacy in boosting grain production efficiency. Therefore, it is recommended that government departments accelerate the ASPS policy and continuously improve the related policy system, ensuring alignment with China’s contemporary grain production landscape. According to the empirical analysis, the following recommendations are advanced.

Firstly, the governments should enhance subsidy investment and policy refinement. The dynamic subsidy allocation adjustment mechanism based on efficiency monitoring results can be established. Regular assessments of policy efficiency outcomes across regions should be conducted, with more resources directed toward areas demonstrating strong policy responsiveness and rapid efficiency gains. Secondly, differentiated ASPS should be implemented to achieve accurate subsidies. In major grain production regions and balance of production and consumption regions, efforts should be intensified to continuously increase investments in ASPS subsidies. In major grain consumption regions, policy expectations and assessment criteria can be adjusted to establish reasonable subsidy ratios and systems, thereby improving grain production conditions. For wheat subsidies, support can be linked to objectives such as “enhancing quality” rather than solely pursuing production efficiency. Thirdly, target core mechanisms by directing subsidy funds to stimulate dual efficiencies in scale and input. On one hand, subsidy policies can be deeply integrated with measures such as land transfer and high-standard farmland construction. For example, farmers who achieve a certain planting scale can be offered additional incentives. On the other hand, subsidy funds should be directed toward productive resources like high-quality seeds and efficient agricultural machinery. This reduces farmers’ costs in adopting cash-based production inputs, ensuring subsidies translate into both qualitative and quantitative improvements in productive resources.

There are some limitations in this study. Firstly, the analysis focuses exclusively on China’s provincial-level implementation of ASPS, utilizing predominantly national statistical sources. Subsequent studies could broaden this scope to municipal units within China or undertake cross-national comparisons of subsidy impacts on agricultural output. Additionally, microdata can be utilized to analyze the differences among farmers of varying scales. Secondly, the mechanism analysis has only explored two aspects of grain cultivation areas and production inputs, so future research could examine the mechanisms from various perspectives. Finally, decomposing TE into PTE and SE enables a more granular interpretation of the sources of efficiency gains. This represents both a limitation of the present study and a direction for future research.

## Supporting information

S1 Data Stata. (XLSX)
